# Effect of Yerba Mate Extract as Feed Additive on Ruminal Fermentation and Methane Emissions in Beef Cattle

**DOI:** 10.3390/ani12212997

**Published:** 2022-10-31

**Authors:** Yuli A. Pena-Bermudez, Rafaela Vincenzi, Paulo Meo-Filho, Leandro S. Sakamoto, Richard Lobo, Gabriela Benetel, Annelise Lobo, Carol Matos, Vanderlei Benetel, Cesar G. Lima, Alexandre Berndt, Laura M. Cardenas, Ives C. S. Bueno

**Affiliations:** 1 Department of Animal Science, Faculty of Animal Science and Food Engineering, University of Sao Paulo (USP), Pirassununga 13635-900, SP, Brazil; 2 Net Zero and Resilient Farming, Rothamsted Research–North Wyke. North Wyke, Okehampton, Devon EX20 2SB, UK; 3 Institute of Animal Science (IZ), Sertãozinho 14160-900, SP, Brazil; 4 Department of Animal Science, Institute of Food and Agricultural Sciences, University of Florida, 2250 Shealy Dr, Gainesville, FL 32608, USA; 5 Brazilian Agricultural Research Corporation (EMBRAPA), Embrapa–Southeast Livestock, São Carlos 13560-970, SP, Brazil

**Keywords:** greenhouse gases, Ilex paraguariensis, natural additive, diet digestibility, nutrient utilization

## Abstract

**Simple Summary:**

The livestock sector contributes a considerable proportion of greenhouse gas emissions. Mechanisms must be developed in this sector to reduce these gases’ emissions. This generates a demand for studies that evaluate plant species or their extracts to define their potential in modulating ruminal fermentation with the objective of reducing enteric methane emissions. The yerba mate extract (YME) is obtained from a plant widely consumed in the southern region of Brazil for its biological properties, with few studies on rumen fermentation, meat quality, and production parameters. For this reason, this study sought to evaluate the inclusion of different levels of YME and analyze its effects on ruminal fermentability, methane emissions, and animal performance. Our results show that the inclusion of YME does not affect ruminal fermentation parameters or apparent digestibility.

**Abstract:**

The inclusion of plant extracts that contain secondary compounds with the potential to modulate rumen fermentation and improve animal performance has gained attention in recent years. The aim of this study was to evaluate the effect of the inclusion of yerba mate extract (Ilex paraguariensis ST. Hilaire) (YME) on the ruminal parameters. Eight castrated cattle were divided into four groups, a control without YME (0%) and three treatment groups with 0.5, 1 and 2% inclusion of YME in the dry matter. The inclusion of YME did not show differences in ruminal methane emissions (CH_4_), and total apparent digestibility (*p* = 0.54). Likewise, YME did not modify ruminal pH, but positively affected NH_3_-N, which decreased linearly as the extract level in the diet increased (*p* = 0.01). No short chain fatty acids (SCFA) were influenced by YME, except isovaleric acid (*p* = 0.01), which showed a lower concentration in the inclusion of 2% YME. Our results show that up to 2% YME does not affect digestibility, ruminal fermentation parameters, or the concentration of short-chain fatty acids in the rumen.

## 1. Introduction

Livestock have been an important component of the total gross domestic product (GDP) in developing countries. For example, livestock production contributed to around 8.1% of total Brazilian GDP [1]. However, the livestock production system contributes to the increase in global warming, emitting considerable amounts of greenhouse gases (GHG), as a result of ruminal fermentation and the decomposition of feces [2] and a source of waste of energy of the diet, which can vary from 2 to 12% of losses of the total diet’ energy [3]. Therefore, the strategic feeding of ruminants is particularly interesting to increase rumen efficiency, improve feed utilization and reduce environmental contamination [4].

Several studies have shown that the use of herbs or plant extracts can manipulate ruminal ecology in terms of improving feed efficiency and reducing CH_4_ emissions [4,5]. An interesting plant candidate as a natural additive for ruminants’ nutrition is the yerba mate (*Ilex paraguariensis* St. Hilaire), a plant widely used in southern Brazil and other countries in South America [6]. Yerba mate is characterized by having a complex chemical composition, found in greater proportion polyphenols (chlorogenic acid) and xanthines (caffeine and theobromine), followed by purine alkaloids (caffeic acid, 3, 4-dicaffeoylquinic acid, 3, 5-dicaffeoylquinic acid), flavonoids (quercetin, kaempferol and rutin), amino acids and minerals [7,8,9]. Previous studies where yerba mate was used in dairy cattle, showed that the inclusion did not affect dry matter intake, milk production or its composition [10]. On the other hand, Po et al. [11], carried out the supplementation of growing lambs, monitoring consumption, live weight and conversion, in addition to antioxidant capacity and wool growth, no significant differences were obtained. In the area of ruminal fermentability, yerba mate supplementation to dairy cows seems to affect the degradation of fiber and protein in the rumen, being able to decrease ammonia production and increase protein availability for productive purposes, given the presence of tannins in the yerba mate composition [12]. In order to obtain conclusive data, the objective of this study was to evaluate the inclusion and effects of yerba mate extract in the diet of Nellore cattle, on digestibility, methanogenesis and fermentation kinetics.

## 2. Materials and Methods

The procedures that use animals were approved by the Institutional Animal Care and Use Committee of the Faculty of Animal Science and Food Engineering of the University of São Paulo (protocol code: 5190160318/2018). The experiment was conducted from 9th April to 4th June 2018 at the cannulated animal sector of the Department of Animal Science of the Faculty of Animal Science and Food Engineering (FZEA) of the University of São Paulo.

### 2.1. Treatments and Experimental Design

Eight castrated rumen-cannulated Nellore cattle (*Bos indicus* L.) steers with initial body weight (LWI) of 401.6 ± 32.3 kg were used. The animals were housed in individual pens of approximately 10 m^2^ with individual access to water and feed. The experimental design used was a replicated contemporary Latin square, with four levels of YME inclusion in the diet: 0, 0.5, 1 and 2% based on DM. The animals were randomly assigned to each of the experimental treatments. The experimental unit was the animal within each Latin square. Thus, the experiment had 32 experimental units referring to 8 animals divided into 2 Latin squares and 4 periods. Each period was comprised of 21 days, consisting of 14 days of adaptation followed by 7 days of sample collection, i.e., the experimental period.

### 2.2. Diet

The animals were fed twice a day at 8:00 h and 15:00 h. The diets were calculated following the nutritional requirements for maintaining beef cattle, according to the NRC, (2000), with a forage:concentrate ratio of 70:30, and following the recommendations of 104 g crude protein and 659 g total digestible nutrients (TDN) per kg of DM. The feed offered and refused were weighed daily to determine the daily DM intake, allowing 10% refusal. Diet samples were collected from day 15 to 21 of each experimental cycle before the diet supply. About 300 g of sample were immediately packed in plastic bags, identified for each animal and experimental period, and frozen at −20 °C. Later, the samples were thawed, placed in identified aluminum trays and put for dry in a forced circulation oven at 65 °C for 72 h. The samples were then homogenized into a composite sample from each animal in each period, ground in a mill with a 1mm sieve, and wrapped in plastic bags for further analysis. The samples were analyzed for AOAC (1990) dry matter (DM, method 934.01), ash (ASH, method 923.03), crude protein (CP, method 920.87) and ether extract (EE, method 920.85). Neutral detergent fiber (NDF) and acid detergent fiber (ADF) determinations were performed according to the methodology proposed by Van Soest et al. (1991). Regarding the gross energy analyses, the content was determined by the complete oxidation of the samples in a calorimetric bomb (C200 System, IKA^®^, Staufen, Germany). The ingredients and chemical composition of the diets are presented in Table 1.

The YME was produced by Centro Flora (Botucatu, SP, Brazil), from fresh leaves of Ilex paraguariensis by water:ethanol 75:25 *v/v* extraction at 90 °C, which were freeze dried until they reached 91.8% dry matter. The extract supplied to the animals in this investigation was the same product previously used by Zawadzki et al. [13], who reported that in its composition the presence of saponins, methylxanthines and phenolic compounds. The most relevant phenolic compounds, expressed in mg g^−1^, in the extract are chlorogenic acid (12.30 ± 0.01), 1, 5-dicaffeoilquinic acid (6.01 ± 0.01), caffeic acid (0.813 ± 0.002), quinic acid (0.877 ± 0.006), shikimic acid (0.0012 ± 0.0001) and gallic acid (0.0179 ± 0.004).

### 2.3. Sampling, Measurements, and Analysis

#### 2.3.1. Methane Sample Collections

The Sulphur hexafluoride tracer gas (SF_6_) technique was used to measure CH_4_ emissions, as described by Johnson et al. [14], evaluated daily for 24 h for five consecutive days in each experimental period (days 16 to 21). Before the CH_4_ sampling, a seven-day adaptation period to the equipment was used, to allow the steers to get used to it. After the adaptation period, the animals were equipped with halters and canisters (PVC U-yokes under vacuum and attached to the neck of the animal). The U-yokes (with an average weight of 1.5 kg) were attached to the animal’s neck using Velcro straps around the U-yokes and halter. Samples (blanks) were also collected at two spots that represented the experimental environment air, and where the animals did not have access to. The CH_4_ and SF_6_ concentrations obtained in the environmental samples were discounted in the calculations of the emissions of the evaluated animals, which was considered as a blank for calculation purposes.

#### 2.3.2. Analysis of Methane Emissions

For the chromatographic analysis, the yokes were pressurized with N_2_ to approximately 10% above atmospheric pressure and the analysis was performed in a Shimadzu model GC-2014 chromatograph (Shimadzu Corporation, Japan) following the method described by Johnson et al. [14]. Calibration curves were established using standard gas certified by “White Martins” (a Praxair Company) with CH_4_ concentrations in parts per million (ppm) (4.85 ± 5%, 9.96 ± 1.65% and 19.1% ± 3.44%), and SF_6_ in parts per trillion (ppt) (34 ± 9.0, 91.0 ± 9.0 and 978.0 ± 98.0), as according to Westberg et al. [15].

The CH_4_ emissions were calculated as the proportion of CH_4_ to SF_6_ in the canister, with each of the gases being adjusted for blank concentration, together with the predetermined permeation rate of SF_6_ capsules, following the same methodology used by Méo-Filho et al. [14]. The CH_4_ emission potential was expressed in different units, namely: kg CH_4_ year^−1^, g CH_4_ day^−1^, kg CH_4_ kg DMI^−1^, and g CH_4_ kg LW^−1^. The percentage of gross energy lost in the form of CH_4_, represent by percentage yield of methane (YM%), is the percentage ratio between the energy lost in the form of methane and the gross energy intake, which was calculated according to Méo-Filho et al. [16].

### 2.4. Fermentation Kinetics

On the 21st day of each cycle, ruminal samples were collected at time 0 (immediately before feeding) and 3, 6, 9 and 12 h after morning feeding. Ruminal content was collected manually from three different points (frontal, median and caudal portion) via the ruminal cannula. The content was then pressed through two layers of cheesecloth for ruminal fluid extraction, which was used to evaluate ruminal fermentation parameters: pH, short-chain fatty acids (SCFA) and ammonia nitrogen (NH_3_-N). Ruminal pH values were determined immediately after collection of the ruminal fluid samples by a portable potentiometer (HANNA Instruments HI 8424, Brazil), calibrated with pH 4.0 and pH 7.0 buffers solutions.

For the SCFA determination, 2 mL of filtered rumen liquor aliquot was collected and packed in a plastic microtube, identified, and stored in a freezer (−20 °C) immediately after collection. The samples were analyzed by gas chromatography (GC-2014, Shimadzu, Japan) using a capillary column (Stabilwax^®^, Restek, Bellefonte, PA, USA) at 145 °C (isothermal) and a split/splitless injector and dual FID detector at 250 °C, according to the method described by Erwin et al. [17] and adapted by Getachew et al. [18]. Helium gas was used as a carrier gas, synthetic air as an oxidizer and hydrogen as fuel. The material was thawed and centrifuged (14500 g for 10 min) and 0.8 mL of supernatant was removed along with 0.2 mL of formic acid (98–100%) and 0.1 mL of the internal standard (100 mM 2-ethylbutyric acid, ChemService, West Chester, PA, USA).

The external standard was prepared with acetic, propionic, isobutyric, butyric, isovaleric and valeric acids (ChemService, West Chester, PA, USA). For the determination of NH_3_-N, 2 mL of the filtered rumen liquor aliquot was collected and stored in an identified glass vials containing 1 mL of 1 N sulfuric acid, corked, and after stored in a freezer until the determination of ammoniacal nitrogen. Analyses were measured by colorimetry using enzymatic urea commercial kit UREIA UV K056 (Bioclin^®^, Belo Horizonte, Brazil), according to the method described by Kulasek et al. [19] and adapted by Foldager [20].

### 2.5. Total Apparent Digestibility

To determine the total apparent digestibility of DM and nutrients, from day 15 to 21, 300 g of the feed refusal was sampled once a day, before morning feeding, within each experimental period. Feces were sampled every 12 h, directly from the animals’ rectum, from day 15 to 19. Thus, on the first day the sampling started at 7:00 am, on the second at 10:00 am, on the third at 1:00 pm and on the fourth day at 4:00 pm to ensure the collection of representative samples for the period [21]. The procedure of collection, storage, drying, grinding and homogenization of feed refusal and feces for further analysis, followed the same protocol for diet samples as described above.

The total feces production of the animals was estimated using indigestible neutral detergent fiber (iNDF) and indigestible acid detergent fiber (iADF) as internal indicators [20]. For this purpose, three castrated and ruminal cannulated Nellore cattle with 524 ± 13 kg initial LW were used for the analysis of iNDF and iADF in the diets, refusal, and feces. These animals belonged to the same group of eight cattle that were subjected to the nutritional experiment, as previously described. The cattle were housed in a paddock, fed twice a day, at 8:00 h and 15:00 h, and received only free corn silage with free access to water, minerals, and pasture. This stage lasted 26 days, of which 14 days were necessary for adaptation to the diets and the last 12 days were used for sample incubation.

To obtain the levels of NDFi and ADFi in the feed, refusal and feces, each pre-dried sample were analyzed in triplicate been placed in non-woven bags (100 g m²^−1^) with dimensions of 5 × 5 cm, according to the ratio of up to 20 mg of dry matter per cm² of surface [21]. The bags were then packed in Raschel nets coupled to a counterweight to ensure that the bags do not float into the rumen. The samples were incubated for 288 h in the rumen of the three animals previously adapted. After removing the bags from the rumen, they were rinsed with running water. Then, the samples were subjected to NDF and ADF analysis in fiber analyzer equipment Mertens, [22] adapted to Tecnal^®^’s recommendations, Piracicaba-SP, Brazil, for 1 h each. After this period, they were washed with hot water and subsequently with acetone, dried in an unventilated oven (105 °C for 4 h), placed in a desiccator and weighed, thus obtaining the iNDF and iADF concentration. The apparent digestibility coefficients of dry matter and nutrients were estimated by Cochran and Galyean [23].

### 2.6. Statistical Analysis

Data were analyzed using the Statistical Analysis System 9.3 package (SAS Inst. Inc., Cary, NC, USA). The presence of outliers and the normality of the residues were tested (Shapiro–Wilk) using the PROC GLM procedure.

Digestibility, nutrient intake and CH_4_ were analyzed using the PROC MIXED procedure, fitting a model that included the fixed effects of treatments (i = 1, …, 4), square (j = 1, 2), and period (k = 1, …, 4) within squares, random effect of animal (i = 1, …, 8) within squares, and the error term. Fermentative kinetics data (pH, SCFA and NH_3_-N) were processed using the PROC MIXED procedure for time-repeated mixed models. The model of these variables included fixed effects of treatment (i = 1, …, 4), time (t = 1, …, 5), interaction between treatment and time, square (j = 1, 2), and period (k = 1, …, 4) within squares, random effect of animal (i = 1, …, 8) within squares, and the error term. The effect of the level of inclusion was analysed by using orthogonal polynomials, separating the effects into linear and quadratic. The coefficients for the trend tests used PROC IML because the inclusion levels were not evenly spaced. Finally, the mean values of the treatments were generated using the LSMEANS option (SAS Inst. Inc., Cary, NC), and compared, if necessary, using Tukey’s test. Statistical significance was declared when *p* < 0.05.

## 3. Results

### 3.1. Apparent Digestibility Coefficients

The DM was not influenced by the extract (Table 2). Similarly, the apparent digestibility coefficients were not influenced by the inclusion of YME in any of the nutrients evaluated (Table 3).

### 3.2. Rumen Methane Production Evaluation

No differences (*p* ≥ 0.58) were observed between the treatments for the CH_4_ emission values (Table 4) expressed in g day^−1^, g kg DMI^−1^, g kg LW^−1^ nor YM%.

### 3.3. Fermentation Kinetics

In general, the inclusion of YME did not affect the production of SCFA between the inclusion levels, in Table 5 the average values of the five times evaluated are presented. The isovaleric acid showed a quadratic effect (*p =* 0.03), and it was also observed a trend of lower production of isobutyric (*p* = 0.1163) and butyric (*p* = 0.0815) acid with increasing the level of YME inclusion.

The pH values were influenced by time (*p* < 0.0001), decreasing linearly with increasing time for the four treatments analyzed (Table 6). On the other hand, the production of SCFA (%) and NH_3_-N in mg dL^−1^ were influenced (*p* < 0.0001) by the times evaluated after feeding (Table 6). This variation over time showed a quadratic effect (*p* < 0.0001), for acetic, isobutyric, valeric, isovaleric, and propionic acids and the acetate: propionate ratio (Table 6), although butyric acid presented variations over time, showed no linear or quadratic effect. For its part, isovaleric acid was the SCFA (%) influenced by diet.

## 4. Discussion

### 4.1. The Apparent Digestibility Coefficients

According to Beauchemin et al. [24], condensed tannin extract used in the proportions 0, 1 and 2% of DM in Angus cattle, fed with a 70:30 forage: concentrate ratio, did not influence nutrient intake in relation to body weight. In this study, the inclusion of YME also did not affect intake variables. A study by Aemiro et al. [25] evaluated the effect of Sunphenon 30S-O, a compound taken from green tea leaves, and demonstrated that nutrient intake was not affected when it was used at low levels (1 to 2.5% of additive per kg of DM). However, when the Sunphenon 30S-O dose was increased to 4% per kg of DM, the intake of DM, NDF and ADF decreased by 15.6% and CP intake decreased by 13.9%. Dschaak et al. [26] also reported reductions in the intake of DM, CP, NDF and ADF in dairy cows ingesting 3% of tannin per kilogram of DM. Probably, nutrient intake is not affected by the low inclusion of extract in the diet. As the inclusion of plant extracts containing tannins and other phenolic compounds increases, the animals linearly decrease their dietary intake [27].

This complex of tannins and proteins is dissociated by the low pH of the abomasum, making proteins available for intestinal absorption [28]. Hartemink et al. [12] reported that yerba mate pellets supplementation in the diet of grazing dairy cows affects fiber and protein degradation inside the rumen, due to the combined processes of solubilization and degradation. However, tannins in feed form complexes with proteins, carbohydrates, and with digestive enzymes, resulting in lower nutrient digestibility [29]. Similar results were reported by Aguiar et al. [30] studying the effects of phenolic compounds present in propolis, who identified positive effects on protein metabolism in the rumen as a result of the inclusion of propolis in diets for dairy cows.

Apparent digestibility coefficients were not affected by the inclusion of the extract. This is a similar result to those reported by Santos et al. [10], who observed that inclusion of dried yerba mate leaves did not affect digestibility of DM, CP and NDF at doses of 250, 500 and 750 g day^−1^ for Holstein cows, but decreased EE digestibility by 2.8%, resulting from the inhibitory effect of yerba mate saponins on lipase activity. The inclusion of phenolic compounds (3.81 mg kg^−1^) from propolis in diets for dairy cows showed higher DM, CP and NDF digestibility compared with inclusion of 1.93 mg kg^−1^ [30], unlike the present study which showed that the 2% extract inclusion tended to decrease the CP and EE digestibility, and the 1 and 2% inclusion tended to decrease the NDF digestibility. Many plants phenolic compounds are polymerized into larger molecules, such as condensed tannins [31], that binds to protein and are regarded as “antinutritional” compounds which reduce protein digestibility [32]. The discrepancies in responses of tannins among different studies are attributed to the different chemical structures and concentrations of tannins, and type of diets [33]. The results observed in this study suggest that increasing the inclusion of YME in the diet to more than 2% could affect nutrient digestibility.

### 4.2. Coefficients Enteric Methane Production

Even though the CH_4_ variables did not show differences among levels of inclusion, there are no studies in the literature that investigated the emission of methane from ruminants receiving YME in the diet. The average CH_4_ emission values in g day^−1^ obtained (234 g day^−1^ animal^−1^) were higher than those reported by Johnson and Johnson [14] for beef cattle (164 to 194 g day^−1^ animal^−1^). However, they are similar to those found by Canesin et al. [34], of 226 g CH_4_ day^−1^ for Nellore cattle with an average live weight of 383 kg and daily DMI of 7.87 kg, fed Urochloa brizantha and a supplement. The same study also reported values of 28.42 g per kg of DMI and 0.59 g of CH_4_ per kg of average live weight were observed, similar to our values of 30.8 and 0.506 g kg^−1^, respectively.

Green tea is an extremely popular plant throughout the world and has medicinal properties. Like yerba mate, it is also rich in phenolic compounds, tannins and saponins. The latter two are known for their capacity to mitigate ruminal methane production [35]. According to Nasehi et al. [36], the partial replacement of alfalfa by green tea leaves in the diets of lambs in an in vitro experiment was responsible for reducing methane emissions from 20.57 ml 200 mg^−1^ DM for the control treatment to 17.18 and 18.01 ml 200 mg^−1^ DM when using 40 and 60 g of green tea per kg instead of alfalfa DM, respectively.

Even though there was no difference observed among the levels of inclusion, the YM% variable is important in determining the amount of raw energy lost in the form of methane with an average accepted value of 6.5% of the gross energy ingested [37]. YM is expressed as a percentage and varies mainly depending on the live weight of the animal and the composition of the diet and can range from 2 to 12% of methane energy loss [14]. The values observed in this experiment were within this range with an average value of 9.6%.

The secondary metabolites present in plants, such as tannins and saponins, can improve the degradation of protein and fiber, and reduce the loss of energy ingested via the emission of enteric methane [38,39]. In their review, Naumann et al. [40] and Min et al. [41] showed the impact of tannins on methanogenesis and the consequent reduction in enteric methane emissions by ruminant animals. The inclusion of phenolic compounds such as caffeic acid in a forage-based diet can inhibit in vitro methanogenesis and rumen fermentation; however, in more concentrated diets, an opposite effect was observed [38]). An explanation for the lack of differences between treatments in the enteric methane variables in the present study is the percentage of inclusion of YME and the restricted amount of dry matter ingested (maintenance).

### 4.3. Fermentation Kinetics

The pH value is a highly relevant variable in ruminal fermentation since it is responsible for maintaining ruminal motility and fiber degradation [42]. Therefore, given the average pH values found in the evaluated groups, it is possible to affirm that the YME does not affect the ruminal pH. According to Ørskov [43], the recommended values for maintaining these conditions are between 6.2 and 7.1, and any change in these values reflects changes in the growth rate of bacteria and protozoa [44], which may have an impact on rumen bacterial communities and consequently in the products of ruminal fermentation, motility and absorption [45].

A pH between 6.0 and 6.2 is indicative of healthy ruminal conditions and values below this favor the growth of lactic acid-producing bacteria [46]. The values found in this study are within the normal range presented by Ørskov [43] and expected for diets with a higher proportion of forage. Some studies evaluating the effect of plant phenolic compounds and tannins, such as green tea, coffee and Oolong tea, demonstrate that these substances are also unable to alter ruminal pH [36]. Ruminal pH is associated with methane emissions in the rumen and SCFA production [47]. These findings support the lack of change in pH among treatments.

According to Satter and Slyter [48], the minimum NH_3_-N to satisfy the growth needs of the microbial population is 5 mg dL^−1^, so the animals in the current study were above the recommended minimum. Similarly, the information reported by Busquet et al. [49] also showed a linear reduction in NH_3_-N values as the concentration of phenolic compounds and tannins and saponins increased in the ruminant diet. This can be explained by the possible formation of complexes between the tannins and saponins in YME and the protein available in the rumen, thus decreasing protein deamination as the extract is added to the diet [50,51].

Just as no differences in methane in methane production reported in this study, SCFA production did not differ, except for isovaleric acid. Isovaleric acid is produced in low amounts, but together with isobutyric acid they are responsible for the growth of microorganisms in the rumen [52]. High concentrations of isovaleric and isobutyric acid favor the assimilation of NH_3_-N by microorganisms, that is to say that the greater proportion of SCFA, the concentrations of NH_3_-N decrease, producing greater numbers of microorganisms in the rumen, reducing the microbial protein to be synthesized by the rumen [53].

## 5. Conclusions

The YME was not able to promote changes in the dry matter intake, apparent digestibility coefficients, enteric methane emissions, or the production of most short-chain fatty acids. Future studies are needed with a greater number of animals, experimental period, and levels of extract inclusion. In order to understand the effects that yerba mate hason animal performance, and whether a greater presence of tannins and phenolic compounds for a longer period can have a different effect on rumen fermentation and thus on enteric methane emission.

## Figures and Tables

**Table 1:** Experimental diets of Nellore cattle fed increasing levels of yerba mate extract.

Variables	Treatments (%)			
	0	0.5	1	2
*Ingredients*, g kg DM^−1^ Corn silage Ground corn Soybean meal Salt Mineral mix ^1^ Yerba mate extract Kaolin ^2^ *Chemical composition*^3^ DM% ASH, % DM CP, % DM EE, % DM NDF, % DM ADF, % DM GE, Mcal kg DM^−1^	700 186 75 8.7 5.3 0 25 50.17 7.53 11.04 3.56 34.94 21.31 4.30	700 186 75 8.7 5.3 5 20 50.17 7.31 11.43 3.63 35.00 21.35 4.32	700 186 75 8.7 5.3 10 15 50.07 6.96 11.12 3.57 34.47 20. 994.34	700 186 75 8.7 5.3 20 5 49.97 6.12 11.38 3.58 34.42 20.93 4.48

^1^ Mineral mix: Minerthal 160 MD^®^, which has the following guaranteed levels per kilogram of product: Ca (min)–208 g, Ca (max.)–218 g, Co–148 mg, Cu–2.664 mg, S–64 g, Fl–1.600 mg, P–160 g, I–141 mg, Mn–2220 mg, Se–37 mg, Zn–7992 mg. ^2^ Kaolin: was used as an inert ingredient to replace the yerba mate extract and maintain a constant nutrient concentration across experimental diets. ^3^ DM: dry matter, ASH: mineral matter, CP: crude protein, EE: ethereal extract, NDF: neutral detergent fiber, ADF: acid detergent fiber, GE: gross energy.

**Table 2:** Dry matter intake of Nellore cattle fed increasing levels of yerba mate extract.

Variables ^1^	Treatments (%)				SE ^2^		*p*-Value ^3^	
	0	0.5	1	2		Diet	Linear	Quadratic
*Intake*								
DM. kg.day^−1^	7.510	7.736	7.831	7.737	0.206	0.6741	0.4504	0.3435
DM. g^−1^ kg LW.day	16.185	16.594	16.906	16.644	0.468	0.6483	0.4355	0.3204
DM. g^−1^ kg LW^0.75^.day	75.092	77.099	78.477	77.222	2.021	0.6425	0.4461	0.3077
CP. kg.day^−1^	0.826	0.892	0.872	0.887	0.025	0.1758	0.1441	0.2681
CP. g^−1^ kg LW.day	1.780	1.912	1.885	1.902	0.054	0.2033	0.1590	0.2246
CP. g^−1^ kg LW^0.75^day	8.257	8.884	8.754	8.832	0.235	0.1954	0.1586	0.2168
EE. kg^−1^ day	0.278	0.29	0.292	0.288	0.008	0.5772	0.4228	0.2837
EE. g^−1^ kg LW.day	0.599	0.622	0.632	0.623	0.197	0.5722	0.3727	0.2878
EE. g^−1^ kg LW^0.75^.day	2.779	2.890	2.928	2.887	0.083	0.5647	0.3888	0.2737
NDF. kg^−1^ day	2.606	2.685	2.677	2.643	0.073	0.8430	0.8398	0.4393
NDF. g^−1^ kg LW.day	5.603	5.752	5.766	5.672	0.172	0.8422	0.8427	0.4092
NDF. g^−1^ kg LW^0.75^.day	26.011	26.732	26.766	26.330	0.744	0.8356	0.8533	0.4040
ADF. kg^−1^ day	1.594	1.645	1.638	1.620	0.045	0.8321	0.8096	0.4439
ADF. g^−1^ kg LW.day	3.427	3.524	3.526	3.468	0.106	0.8391	0.8072	0.4223
ADF. g^−1^ kg LW^0.75^.day	15.909	16.379	16.372	16.139	0.458	0.8306	0.8183	0.4139

^1^ LW: Liveweight, DM: Dry matter, EE: Ether extract, NDF: Neutral detergent fiber, ADF: Acid detergent fiber. ^2^ SEM: Standard error of the mean. ^3^ Linear: Linear probability effect, Quadratic: Square probability effect.

**Table 3:** Apparent nutrient digestibility coefficients of diets with levels of yerba mate extract fed to Nellore cattle.

Variables (%) ^1^	Treatments (%)				SEM ^2^		*p*-Value ^3^	
	0	0.5	1	2		Diet	Linear	Quadratic
DMD	66.8	66.7	66.4	65.7	0.008	0.7376	0.2856	0.8741
CPD	65.8	66.9	65.2	64.1	0.009	0.2003	0.0881	0.4912
EED	79.6	80.2	80.4	76.3	0.012	0.1185	0.0553	0.1332
NDFD	43.3	43.5	41.8	41.0	0.011	0.2603	0.0737	0.9576
ADFD	40.8	42.8	40.8	40.7	0.011	0.3489	0.5423	0.5108

^1^ DMD: Dry matter digestibility, CPD: Crude protein digestibility, EED: Ether extract digestibility, NDFD: Neutral detergent fiber digestibility, ADFD: Acid detergent fiber digestibility. ^2^ SEM: Standard error of the mean. ^3^ Linear: Linear probability effect, Quadratic: Square probability effect.

**Table 4:** Methane emissions by Nellore cattle fed increasing levels of yerba mate extract.

Variables ^1^	Treatments (%)				SEM ^2^	*p*-Value ^3^		
	0	0.5	1	2		Diet	Linear	Quadratic
CH_4_GD, g day^−1^	230	238	238	237	8.94	0.7648	0.5417	0.5276
CH_4_DMI, g kg^−1^	30.8	31.0	30.8	30.6	0.94	0.9861	0.8313	0.8604
CH_4_LW, g kg^−1^	0.497	0.509	0.514	0.505	0.15	0.8489	0.7729	0.4148
YM, %	9.60	9.60	9.50	9.50	0.28	0.9783	0.6838	0.9284

^1^ CH_4_GD: Methane emission in g per day, CH_4_DMI: Methane emission in g per kg of dry matter intake, CH_4_LW: Methane emission in g per kg bodyweight, YM,%: Percentage of gross energy in feed converted to methane. ^2^ SEM: Standard error of the mean. ^3^ Linear: Linear probability effect, Quadratic: Square probability effect.

**Table 5:** Average short-chain fatty acid production of Nellore cattle fed increasing levels of yerba mate extract.

Variables (%)	Treatments (%)				SEM ^1^	*p*-Value ^2^		
	0	0.5	1	2		Diet	Linear	Quadratic
Acetic	62.91	62.47	63.27	62,81	0.1594	0.7474	0.8954	0.8080
Propionic	18.05	19.09	19.41	19.41	0.1972	0.4668	0.2082	0.3578
Isobutyric	1.28	1.3	1.31	1.23	0.0166	0.1403	0.1163	0.0812
Butyric	14.66	13.98	13.01	13.66	0.1211	0.0754	0.0815	0.0559
Isovaleric	1.82	1.871	1.82	1.60	0.0257	0.0015	0.0005	0.0304
Valeric	1.29	1.30	1.25	1.30	0.0175	0.8076	0.9203	0.5566
Acet: Prop *	3.55	3.32	3.33	3.33	0.0407	0.6183	0.3927	0.4018

* Acetate: Propionate ratio. ^1^ SEM: Standard error of the mean. ^2^ Linear: Probability of linear effect; Quadratic: Probability of square effect.

**Table 6:** Short chain fatty acids, pH, and NH_3_-N production average from Nellore cattle fed increasing levels of yerba mate extract at five different times.

Time (h)	Treatment (%)	Variables								
		pH	Acetic	Propionic	Isobutiric	Butyric	Isovaleric	Valeric	Acet:Prop ^3^	N-NH_3_ (mg dL^−1^)5 ^4^
0	0	7.00	64.83	16.55	1.34	14.32	1.96	0.97	3.97	9.21
	0.5	6.94	63.92	17.32	1.37	14.31	2.02	1.06	3.73	11.2
	1	7.04	65.27	17.49	1.41	12.78	2.06	0.99	3.75	8.31
	2	6.93	64.60	17.41	1.31	13.75	1.85	1.07	3.76	10.51
3	0	6.68	61.98	18.31	1.25	14.98	2.01	1.45	3.41	15.59
	0.5	6.64	62.20	18.87	1.26	14.19	2.01	1.46	3.32	14.13
	1	6.69	63.08	18.97	1.25	13.33	1.97	1.39	3.34	9.74
	2	6.67	62.75	18.94	1.20	14.00	1.70	1.40	3.37	10.22
6	0	6.70	62.65	17.78	1.36	15.16	1.80	1.24	3.56	9.20
	0.5	6.77	62.69	18.51	1.35	14.40	1.86	1.19	3.42	10.02
	1	6.74	63.48	18.67	1.31	13.51	1.82	1.20	3.42	7.93
	2	6.67	62.55	19.34	1.26	13.94	1.60	1.29	3.32	9.16
9	0	6.31	61.94	19.28	1.32	14.15	1.78	1.53	3.25	16.26
	0.5	6.35	61.39	20.62	1.37	13.24	1.85	1.52	3.00	14.73
	1	6.31	61.74	20.67	1.39	12.86	1.82	1.52	3.00	13.83
	2	6.31	61.63	21.08	1.25	13.06	1.49	1.48	3.00	13.17
12	0	6.25	63.15	18.31	1.12	14.66	1.55	1.21	3.54	5.68
	0.5	6.25	62.13	20.11	1.15	13.74	1.61	1.25	3.12	7.97
	1	6.32	62.32	20.43	1.11	13.31	1.61	1.22	3.08	6.59
	2	6.44	62.50	20.24	1.10	13.57	1.34	1.24	3.21	5.75
SEM ^1^		0.06	0.61	0.75	0.07	0.50	0.10	0.05	0.15	1.21
*p-Value* ^2^										
Diet		0.9665	0.7474	0.4688	0.1403	0.0754	0.0015	0.8076	0.6183	0.0637
Time		<0.0001	<0.0001	<0.0001	<0.0001	<0.0001	<0.0001	<0.0001	<0.0001	<0.0001
Diet vs. Time		0.2093	0.2913	0.4096	0.9707	0.9668	0.7664	0.6231	0.3130	0.2985
Linear		<0.0001	<0.0001	<0.0001	0.0018	0.3018	<0.0001	<0.0001	<0.0001	0.0129
Quadratic		0.2546	<0.0001	<0.0001	<0.0001	0.0782	0.0362	<0.0001	<0.0001	<0.0001

^1^ SEM: Standard error of the mean. ^2^ Linear: Probability of linear effect. Quadratic: Probability of square effect. ^3^ Acet: Prop: acetate: Propionate ratio. ^4^ NH_3_-N (mg dL^−1^): Ammonia nitrogen.

## Data Availability

All authors ensure that all data and materials support the findings and comply with field standards. The data presented in this study are available on request from the corresponding author.
